# Eye-Strain in Health and Disease

**Published:** 1897-08

**Authors:** 


Eye-Strain in Health and Disease. With special reference to the Amelioration or Cure of Chronic Nervous Derangements Without the Aid of Drugs. By Ambrose L. Ranney, A. M., M. D., Author of "Lectures on Nervous Diseases," "The Applied Anatomy of the Nervous System," etc., etc.; late Professor of Nervous Diseases in the Medical Department of the University of Vermont and of the Anatomy of the Nervous System in the New York Post-Graduate Medical School, etc. Illustrated with 38 wood-cuts. One volume, royal octavo, pages viii, 321. Extra cloth, beveled edges, $2, net. The F. A. Davis Co., Publishers, 1914 and 1916 Cherry Street, Philadelphia; 117 W. Forty-second Street, New York; 9 Lakeside Building, Chicago.

This is a book which should arrest the attention of every practitioner of medicine, and should be diligently studied by him, whether he makes a specialty of eye treatment or not.

It relates particularly to the correction of defects of vision with relation to the reflexes which such errors of vision, if remaining uncorrected, either provoke or aggravate. The reflexes particularly considered are headache, neuralgia, St Vitus' dance, sleeplessness, epilepsy. nervous prostration, and insanity. If it seem that the claims of the author are extravagant and those of one who is riding a hobby, it may be said that the reader should carefully study the pages of the book for himself and so have the impression dispelled.

The great recommendation of the book, the one which has induced the writer of this review to say at the beginning of this article that it should be diligently studied by every practitioner, is that it is designed especially for beginners. In our opinion it fulfills this mission admirably. It presupposes no knowledge of the means for measuring errors of accommodation; it takes for granted no technical knowledge except that of anatomy, but proceeds to explain simply the most trivial details of lenses, prisms, scales of measurement, instruments of precision for making the measurements, and the exact steps necessary in determining the character of the abnormality, whether of refraction or of the muscles of the eye-ball. In perusing the pages and making himself familiar with the best methods of eye examination, the reader is desired by the author of the book to "see in them no affectation of science or pretense to superior knowledge, but an honest and earnest purpose to bring to notice, in a manner that is simple and practical, a statement of clinical facts that are to-day receiving no small amount of attention from scientific medical men, and a theory that is based upon nature and common sense."

The author asks the question, "Can nervous diseases be caused by eye strain?" He then proceeds to answer this question affirmatively all through the pages of the book.

He is an advocate of the early use of glasses after careful investigation by the methods laid down in the books of the exact nature of the defective sight. He combats the usual opinion that glasses should not be resorted to as long as it is possible to do without them because a person is supposed to become too dependent upon them by a counter proposition: "Because nature becomes dependent upon a glass which gives relief and corrects an existing strain upon the eye, no time should be lost in affording this relief." He illustrates his position by asking a question upon a comparison: "Should a hip splint be avoided when the pain in the joint is arrested by it because the patient feels his dependence upon the splint?"

He then proceeds to describe the various test glasses, and gives wood-cuts of the sets and of the test types and of all the beautiful and exact apparatus required in making measurements. The description is lucid and yet close, and the beginner can very quickly learn to use test glasses just from the descriptions of the book. At page 35 he gives a table of the different errors of refraction and of muscular adjustment of the eye in the orbit which is very instructive, as it shows the relationship of these abnormalities at a glance. We are almost tempted to reproduce this table, and refrain only from want of space.

Beautiful apparatus is described and illustrated designed to show the movements of the eye-balls, and so of determining any unbalanced action of the orbital muscles.

The author appears to have made a specialty of the determination of heterophoria or abnormal adjustment of the muscles rotating the eye-ball. He therefore lays great stress upon the effects of this particular group of abnormalities, and of the method of discovering their presence in any patient and bringing about correction.

Heterophoria, in the very wide and remarkably striking experience of the author, plays a stupendous part in the causation of such diseases as chorea and epilepsy. According to the author this abnormality creates a continual and unconscious expenditure of nervous energy on the part of the patient to overcome it and get normal vision—nervous leakage the author calls it. This leakage deranges the nervous system and brings about these shocking neuroses, accordingly as the patient is predisposed to one or the other.

He corrects the refractive troubles with glasses, and overcomes the abnormal action of the eye muscles by "graduated tenotomies." He relates cases cured or benefited by these procedures to back up his claims. His "case sixth" of epilepsy cured in this way is one of the most remarkable records ever contributed to medical annals.

It should be stated that the author is not so much an eye specialist as a nerve specialist, and therefore all these corrections of eye strain are subservient to a much deeper and broader motive than merely enabling a person to see well. This item lends an additional interest to what he teaches upon the subject of eye strain.

His "graduated tenotomies" are minutely described, though it would not be well for a beginner to attempt to perform them without previous practical training.

Much of the detail of these operations of tenotomy are due, according to the author, to Dr. George T. Stevens, whom the reader will remember as at one time the American editor of *Annales d'Oculistique*, reviewed in THE HOMŒOPATHIC PHYSICIAN for 1896, pages 56, 453, and 491. Enough has here been said; now let every reader procure the book and study it carefully for himself.

